# The Prophylactic and Therapeutic Use of the Heli-FX EndoAnchor System in Patients Undergoing Endovascular Aortic Aneurysm Repair—A Scoping Review

**DOI:** 10.3390/medicina62010040

**Published:** 2025-12-25

**Authors:** Konstantinos Dakis, George Apostolidis, Petroula Nana, George Kouvelos, Eleni Arnaoutoglou, Athanasios Giannoukas, Miltiadis Matsagkas, Konstantinos Spanos

**Affiliations:** 1 Vascular Surgery Department, University Hospital of Larissa, 41334 Larissa, Greece; kostasdakis1994@gmail.com (K.D.); geokouv@gmail.com (G.K.); agiannoukas@hotmail.com (A.G.); milmats@gmail.com (M.M.); spanos.kon@gmail.com (K.S.); 2 German Aortic Centre, Department of Vascular Medicine, University Medical Centre Eppendorf, 20251 Hamburg, Germany; apgiorgos@yahoo.gr; 3 Anesthesiology Department, University Hospital of Larissa, 41334 Larissa, Greece; earnaout@gmail.com

**Keywords:** Heli-FX Endoanchors, EVAR, endoleak, reintervention, aortic neck

## Abstract

*Background and Objectives*: Proximal aortic neck-related complications severely impact the short- and long-term durability of endovascular aneurysm repair (EVAR). The Heli-FX EndoAnchor system provides proximal sealing zone reinforcement, aiming at both prevention and treatment of endograft migration and type Ia endoleak (EL Ia). The aim of this scoping review was to accumulate data on the prophylactic and therapeutic effect of EndoAnchors on patients undergoing index and revision EVAR for proximal neck complications. *Methods and Materials*: The PRISMA Extension for Scoping Reviews (PRISMA-ScR) Guidelines were followed. The literature published between 1 January 2009 and 1 September 2025 was searched by two independent reviewers. Studies reporting on morphological and clinical outcomes related to the proximal aortic neck were included. Main outcomes were Heli-FX EndoAnchor system technical success and procedural EVAR success, aortic neck dilation, endograft migration, EL Ia and proximal neck reinterventions. *Results*: Sixteen studies were included, with a total of 1164 patients. The mean follow-up ranged between 7 and 60 months. Eleven studies provided data on hostile proximal neck characteristics indicating Heli-FX EndoAnchors deployment. Technical success for prophylactic and therapeutic Heli-FX EndoAnchor application ranged between 85 and 100% as well as 86 and 100%, respectively. Procedural success for index and revision EVAR ranged between 85 and 100% as well as 45.4 and 100%, respectively. Residual EL Ia was reported in 103 patients following EndoAnchors deployment during index EVAR and revision cases. Secondary reinterventions related to the proximal sealing zone were reported in 39 patients (17 index EVAR, 19 revision). Mean aortic neck diameter increase between 2.5 and 4.6 mm was reported in four studies, while one study reported a mean >5 mm decrease. *Conclusions*: The Heli-FX EndoAnchor system was associated with high technical success, while procedural success was acceptable, amendable to neck-related characteristics, especially in revision cases for EL Ia treatment. Long-term data on morphological and clinical outcomes are warranted.

## 1. Introduction

Endovascular aortic aneurysm repair (EVAR) has transformed abdominal aortic aneurysm (AAA) treatment by reducing the perioperative morbidity and mortality relative to open surgical repair [1,2]. However, hostile proximal neck characteristics, including short length, conicity, large diameter, severe angulation or heavy calcification, increase the risk of aortic neck dilation (AND) and consequently, endoleak (EL) Ia formation, device migration, and late reintervention [3,4]. The Heli-FX EndoAnchor system (Medtronic, Santa Rosa, CA, USA) is the first endoluminal fixation system introduced as a prophylactic measure during index EVAR in patients with hostile neck characteristics, as well as a minimal therapeutic approach in patients presenting with EL Ia or endograft migration after failed EVAR.

Pooled systematic review data and reports from the multicenter ANCHOR registry showed that EndoAnchors can achieve high technical success and low short-term rates of migration and reinterventions when the Heli-FX EndoAnchor system is used either as prophylaxis or therapeutic measure [5,6,7]. Subsequent focused analyses on patients with AAA and hostile proximal neck characteristics similarly reported low migration and EL Ia rates during the 12-month follow-up [8]. However, a successful Endoanchor penetration is less likely in wide or heavily calcified proximal aortic necks and may be associated to an increased risk for EL Ia [9]. Moreover, longitudinal analyses of neck diameter after index EVAR with concomitant EndoAnchor placement suggested that AND can still occur, raising uncertainty about the definite prophylactic effect of the system [10].

This scoping review aimed at the evaluation of the existing literature on the prophylactic and therapeutic use of the Heli-FX EndoAnchor system, evaluating clinical outcomes and morphological changes related to the proximal aortic neck.

## 2. Materials and Methods

### 2.1. Eligibility Criteria

**Figure 1:**
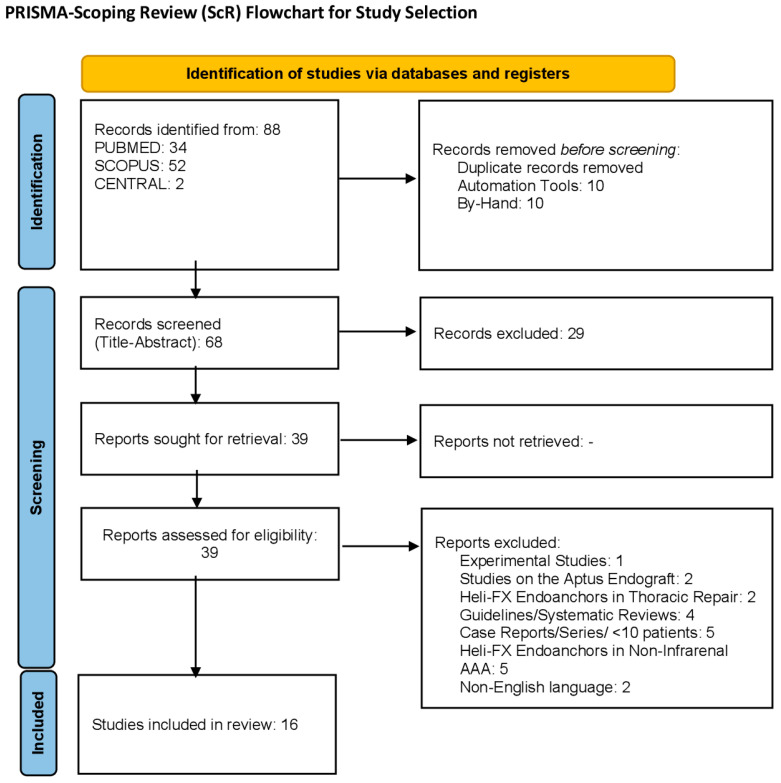
The PRISMA flowchart presenting the study selection process.

The PRISMA Extension for Scoping Reviews (PRISMA-ScR; Figure 1) Guidelines were followed (Supplementary Table S3) [11]. A data search of the English literature was performed using the MEDLINE and EMBASE databases, with a timeframe set between 1 January 2009 and 1 September 2025 given that the Heli-FX EndoAnchor system was initially used in the clinical setting in 2011, outside of the phase 1 STAPLE-1 trial [12].

The P.I.C.O. model (Supplementary Table S1) was applied for providing a clinically guided, specific search strategy [13]. The main inclusion criterion for studies was to report on morphological characteristics and technical outcomes of patients who underwent EVAR for an infrarenal AAA or for the treatment of device migration and/or EL Ia with the adjunctive use of the Heli-FX EndoAnchor system. Morphological characteristics included proximal aortic neck diameter, volume, calcification, thrombus, conicity, angulation, and further, AND. Technical outcomes included any device migration and EL Ia event.

Exclusion criteria included case reports, technical notes, case series, experimental studies on non-human subjects, studies with less than 20 patients, reviews, metanalyses, letters to the editor, and editorials.

### 2.2. Search Strategy

The terms “abdominal aortic aneurysm”, “AAA”, “infrarenal abdominal aortic aneurysm”, “endovascular aortic aneurysm repair”, “endovascular aneurysm repair”, “EVAR”, “Heli-FX EndoAnchor”, “Aptus EndoAnchors”, “HeliFX EndoAnchors”, “endoleak Ia”, “EL Ia”, “device migration”, “endograft migration”, “aortic neck dilatation”, and “proximal neck dilatation” were used in various combinations, both as specific items and MeSH Terms (Supplementary Table S2) [14]. Duplication screening was executed via automated tools (EndNote 20.2.1 X9, Clarivate). A primary selection of relevant studies was based on title and abstract and was performed by two investigators (K.D. and G.A.). A secondary selection was performed according to the full text of publications (K.D. and G.A.). Discrepancies at any step of the process were resolved after discussion with a third investigator (K.S.) References of the included studies were subsequently screened for possibly missing articles [14].

### 2.3. Data Extraction

Data extraction and retention was undertaken on a standard Microsoft Excel spreadsheet. Data relevant to the predefined clinical questions (Supplementary Table S2) were extracted, including also general study information (authors, study title, journal, year of publication, study type, number of included centers), patient-specific infrarenal aortic aneurysm characteristics (maximum sac diameter, proximal neck characteristics such as diameter, volume, calcifications, thrombus, conicity, angulation), and treatment-specific details (type of endograft deployed, number and orientation of Heli-FX EndoAnchors, deployment during index EVAR or as an adjunct for EL Ia treatment). Outcome extraction focused on morphological alterations of the aortic neck following the application of the Heli-FX EndoAnchor system as prophylactic (index EVAR with concurrent Heli-FX endoanchors) or therapeutic (revision EVAR for EL Ia and/or endograft migration treatment using the Heli-FX EndoAnchor system) measure. Any new EL Ia, device migration, and AND was recorded.

### 2.4. Definitions

Technical success related to the Heli-FX EndoAnchor was defined as the deployment of the desired number of EndoAnchors with adequate aortic wall penetration, without any associated EndoAnchor fracture [15]. As per the definition in the initial ANCHOR registry, procedural success was defined as EVAR’s technical success without EL Ia in the completion angiography [15]. According to the reporting standards, EL Ia is defined as the loss of seal at the proximal zone resulting in blood flow towards the aneurysm sac [16,17]. Device migration was defined as ≥10 mm caudal movement of the endograft in relationship to the anatomical landmarks compared with the baseline (1st available post-operative) imaging or any movement <10 mm associated with loss of proximal sealing, EL Ia or increased risk of rupture [16,17]. In case the predefined terms were not used for reporting outcomes, a thorough evaluation of the each described outcome was undertaken.

### 2.5. Risk of Bias Assessment

Risk of bias evaluation was addressed via the ROBINS-I tool for observational, non-randomized studies [18]. Studies were evaluated bearing a “low”, “moderate”, “serious”, or “critical” risk of bias based on a 7-domain assessment model. Each domain addresses sections of possible bias, thus providing a “low”, “some concerns”, or “high” risk of bias score. Risk of bias assessment was carried out by two independent investigators (K.D., G.A.). In cases of discrepancy, a third, senior investigator was advised (K.S.).

### 2.6. Data Analysis

A descriptive report of the outcomes was performed due to the nature of the review and the expected heterogeneity of the reported data.

### 2.7. Ethics

This review complied with the declaration of Helsinki. Scientific Council approval was not required.

## 3. Results

### 3.1. Study Selection

The initial search yielded 88 studies. Following duplicate removal, 20 studies were excluded (10 via automated duplicate removal, 10 via hand duplicate removal). Following title and abstract screening, 29 studies were excluded. Full-text scrutiny excluded 21 studies, producing 18 studies which were evaluated for data overlap. Finally, 16 studies were included in final qualitative analysis; 5 prospective and 11 of retrospective design [8,10,15,19,20,21,22,23,24,25,26,27,28,29,30,31]. Eight studies were produced via a multicenter trial. Reports from two registries were included in the final review (ANCHOR, PERU).

Data overlap was recognized in three cases: six studies [7,8,9,15,21,28] presented results from the ANCHOR registry, with two pairs producing similarly reported results, leading to the exclusion of two studies [7,9]. Three studies produced results from the PERU registry, albeit regarding different follow-up periods, thus none was excluded [23,26,30]. Two studies by Chaudhuri et al. produced results possibly from the same cohort of patients, albeit in different time periods, thus none were excluded [10,25] (Table 1).

### 3.2. Study Population

Five studies included both intact and ruptured AAA patients (619 intact, 17 ruptures) [8,22,24,25,27]. Nine studies included cohorts where the Heli-FX EndoAnchor system was deployed both as prophylactic (671 patients—index EVAR) and therapeutic (217 patients—EL Ia, or endograft migration) measure [8,10,21,22,23,25,27,30,31] (Table 2).

### 3.3. Morphological Features

Regarding indications for EndoAnchor deployment, especially during index EVAR, eleven studies specifically reported on the proximal aortic neck characteristics, focusing on length, diameter, infrarenal angulation, and conical configuration (tapered, reverse tapered), as well as on the presence of circumferential calcifications and mural thrombus [10,15,20,21,22,23,26,27,29,30,31]. In these studies, neck length < 10–15 mm, neck diameter >28–29 mm, and infrarenal angulation > 45–60^°^ were considered hostile neck characteristics [10,15,20,21,22,23,26,27,29,30,31] (Table 3).

All but one study reported on the type of endograft that was deployed in conjunction with the Heli-FX EndoAnchors during the index EVAR procedure [8,10,15,20,21,22,23,24,25,26,27,28,29,31]. Fifteen studies reported on the mean number of Heli-FX EndoAnchors deployed per procedure, ranging from 3 up to 12 [8,10,15,20,21,22,23,24,25,26,27,29,30,31] (Table 4).

### 3.4. Technical Outcomes

The mean follow-up duration was reported in all studies, ranging from 7 to 60 months. Fourteen studies reported on the technical success of either prophylactic or therapeutic Heli-FX EndoAnchor application. Technical success ranged between 85% and 100%; from 85% to 100% for prophylactic/index and 89% to 100% for therapeutic/revision cases. Residual EL Ia following EndoAnchor deployment was reported in 103 patients, across 15 studies; 49 EL Ia were reported following index EVAR procedures, 28 EL Ia were reported following revision procedures, while data were not available for the 26 remaining cases (Table 5).

Procedural success was reported in 13 studies, ranging from 45.4% to 100% in therapeutic cases and from 85% to 100% for prophylactic procedures. At maximum follow-up for each respective study, a new postoperative EL Ia was reported in six patients. During maximum follow-up, freedom from EL Ia ranged between 89% and 100% for index EVAR, while it was reported as low as 77% in revision procedures. Secondary proximal aortic neck reinterventions were reported in 9 studies and 39 patients. Reinterventions following index and revision EVAR was noted in 17 and 19 patients, respectively, while data could not be extracted for 3 patients. Reinterventions were associated with EL Ia treatment in 31 patients (79.5%) and device migration without evident EL Ia in 1 patient (2.5%). Data could not be extracted in seven patients.

Regarding proximal aortic neck diameter, seven studies presented relevant data [10,19,20,21,23,25,27]. The proximal aortic neck diameter showed a mean increase between 2.5 and 4.6 mm in four studies [10,20,21,25], a mean decrease > 5 mm in one study [19] and remained stable in two studies [23,27] (Table 6).

### 3.5. Quality Assessment

All studies were attributed a “serious” risk of bias score, following systematic scrutiny based on the domains of the ROBINS-I tool [18]. Major factors contributing to the score were the confounding factors not reported in the included studies (number of surgeons, expertise, configuration of Endoanchor placement), the ambiguity in the selection of patients with hostile neck characteristics (presence of mural thrombus and/or calcification), lack of a clearly reported, stratified follow-up surveillance strategy and systematic reporting of proximal neck-related outcomes (aortic neck dilatation, device migration) (Supplementary Figure S1).

## 4. Discussion

The Heli-FX EndoAnchor system has been commercially available for more than a decade and has been used as a prophylactic measure to index EVAR procedures, especially in patients with hostile anatomy, as well as a therapeutic tool during revision interventions in patients with previous failed EVAR, due to device migration and EL Ia [12,28,31]. The literature suggests that EndoAnchors have consistently reported high technical success rates, reaching 100%, regardless the underlying pathology or the presence of a failed endograft [7,8,20]. Data derived from large registries (ANCHOR, PERU) demonstrated the positive effect of the Heli-FX EndoAnchor system when standard device fixation is considered inadequate based on preoperative proximal aortic neck characteristics [7,27]. Challenging aortic neck morphology, including short neck length (<10–15 mm), increased neck diameter (>28 mm), high infrarenal β angulation (>60^°^), and conical neck configuration (tapered, reverse tapered, hourglass, barrel shaped, bulged), is the main factor that leads a decision towards a prophylactic EndoAnchor placement, while device migration with or without EL Ia guides its use in revision cases [8,9].

As per the instructions for use (IFU) of the device, the implantation of EndoAnchors in the presence of mural thrombus or diffuse calcification >2 mm in thickness may be associated with implantation difficulties and suboptimal endograft fixation and sealing. Seven studies incorporated in the current analysis reported the inclusion of patients with such proximal neck characteristics, without distinct reporting of the number of patients for each characteristic or specific details on proximal neck’s features (distribution and thickness) [15,21,22,23,26,30,31]. Inadequate proximal sealing and fixation could attribute worse perioperative and long-term outcomes, with procedural success in these studies reported as low as 87% [21]. The number and configuration of EndoAnchors deployed is also crucial for optimal sealing and fixation, with a recommendation for at least four and six in proximal aortic diameters < 29 mm and >29 mm, respectively. While most studies reported deployment of at least four EndoAnchors across their patient population, their clock position within the proximal aortic neck was not clearly described, except for one study [21]. Meticulous preoperative imaging evaluation and planning should guide the optimal position for EndoAnchor implantation according to the IFU to reassure favorable outcomes [32].

Both single-center and multicenter reports highlight the association between postoperative technical and clinical success and a number of parameters, including standardization of operator technique and proper device selection [15,23,24]. Prophylactic deployment of EndoAnchors during index EVAR seems to provide good proximal fixation of the endograft to the aortic wall with immediate apposition [15,23,24,31]. EL Ia rates during index EVAR without the deployment of EndoAnchors ranges between 1 and 2% in non-hostile neck anatomies and 2.5 and 5% in hostile neck anatomies [32]. Bailout deployment for intraoperative EL Ia management and postoperative EL Ia treatment seems to provide a less effective seal, with an EL Ia rate of 7–10% during the early 30-day follow-up [15,28].

Early follow-up imaging after EndoAnchor application suggested good apposition of the device onto the aortic wall, attributed mainly to the sealing properties of the Heli-FX EndoAnchor system, which simulates a hand-sewn anastomosis [7,21,24]. Endograft stabilization to the adjacent aortic neck is associated with reduced rates of device migration, without though necessarily providing positive structural changes of the proximal aortic neck anatomy [10,22,26]. Continuous AND and prevalence of acute infrarenal β angulation regardless of prophylactic or therapeutic use of EndoAnchors has been reported during the midterm follow-up [10,22,26,27,31]. EndoAnchor deployment in revision cases seems to promote endograft stabilization and sealing in hostile neck anatomies with definite presence of EL Ia, although the findings regarding the morphological and structural aortic neck remodeling are conflicting among the available studies [24,25,27,33]. This heterogeneity could be partially attributed to preoperative aortic wall quality at the level of placement, in addition to hemodynamic and genetic factors, related to a more aggressive progression of the disease [26,27,33].

Long-term outcomes in terms of durability, including freedom from EL Ia, and clinically significant device migration are deemed acceptable based on reports from multicenter registries [26,31]. Data from the included studies report on freedom for EL Ia rate ranging from 84% up to 100%, with lower rates reported in studies where the HeliFX EndoAnchor system was therapeutically used for EL Ia treatment (Table 5). Five-year outcomes from the ANCHOR registry suggested promising sac regression rates of 68.2% and acceptable EL Ia rate (12.0%), especially when taking into consideration the application of the Heli-FX EndoAnchor system on previously failed EVAR, a factor related to increased technical complications even when other more complex solutions are applied (e.g., proximal extension using fenestrated endovascular aortic repair after failed EVAR), which target landing into more proximal aortic segments [28,32]. Reports from the ANCHOR and PERU registries suggest low long-term reintervention rates for recurrent EL Ia and progressive AND [28,31]. Meta-analyses corroborate these findings, regardless of the heterogeneous study designs of the included literature, which suggests that diligent postoperative imaging is crucial especially in cases with progressive AND and eminent EL Ia, where endovascular modalities such as fenestrated and branched devices could provide more robust treatment options [6,34].

The ESVS guidelines on AAA repair recommend that for high-surgical-risk patients with hostile proximal neck characteristics where standard EVAR cannot safely achieve proximal sealing, a fenestrated or branched repair (F/BEVAR) should be recommended as first-line modality [35]. Considering that the literature on EndoAnchors is confined in industry-sponsored analyses, in addition to the fact that the only available long-term (>5-years) follow-up is derived from the ANCHOR registry, the guidelines recommend the use of endostapling systems only under a clinical trial setting for patients with hostile neck anatomy in whom F/BEVAR is not an option [35]. However, patient selection and individualized approach seem to be mandatory.

F/BEVAR has been associated with reinterventions attributed mainly to the target vessel instability during follow-up, and patients at high surgical risk presenting with hostile target vessel anatomy for F/BEVAR may be considered as candidates for other solutions, including EndoAnchors [27]. Additionally, specific patient groups, including female patients, carry higher risk for hostile neck anatomy, often requiring a more aggressive proximal neck approach than standard EVAR [36,37]. Considering that the available literature suggests worse postoperative outcomes for female patients undergoing complex endovascular aortic repair, EndoAnchors may provide a valuable alternative, as they require shorter aortic coverage, lower operation times, less renovisceral vessel manipulation, and lower device profile while providing clinically significant proximal neck remodeling and protection towards EL Ia [37,38,39,40].

### Limitations

The retrospective nature of the majority of the included studies negatively affects the quality of the reported pooled outcomes. Additionally, the lack of consensus between definitions of important parameters when reporting neck-related outcomes such as AND and device migration-specific thresholds increases heterogeneity among studies. Limited randomized data and variable imaging protocols also hinder the quality of the produced outcomes. Association between specific anatomic neck characteristics and postoperative outcomes was scarcely reported. Finally, the absence of postoperative neck characteristic evaluation following EndoAnchor placement, mainly diameter, limits statistical and quantitative data analysis.

## 5. Conclusions

The Heli-FX EndoAnchor system is a valuable adjunct for proximal aortic neck complication prevention and treatment. Technical failures are rare and procedural success was acceptable, although amendable to neck-related characteristics. Its effect on postoperative proximal neck morphology should be further examined as data, while promising, is scarce.

## Figures and Tables

**Table 1:** Study Characteristics.

Author	Journal	Publication Year	Study Period	Type of Analysis	Number of Participating Centers
Avci et al. [19]	*J Cardiovasc Surg*	2012	2010–2011	Prospective	1
Perdikides et al. [20]	*J Endovasc Ther*	2012	2010–2012	Prospective	2
Jordan Jr WD et al. [15]	*J Vasc Surg*	2014	2012–2014	Prospective	43
Jordan Jr WD et al. [8]	*Vascular*	2015	2012–2014	Prospective	43
Goudeketting et al. [21]	*J Endovasc Ther*	2019	2010–2017	Retrospective	1
Giudice et al. [22]	*JRSM Cardiovasc Dis*	2019	2015–2018	Retrospective	1
Valdivia et al. [23]	*Ann Vasc Surg*	2019	2011–2017	Prospectively Collected, Retrospective Analysis	4
Ho et al. [24]	*Ann Vasc Surg*	2020	2016–2018	Prospectively Collected, Retrospective Analysis	1
Chaudhuri et al. [10]	*Cardiovasc Intervent Radiol*	2020	2013–2019	Prospectively Collected, Retrospective Analysis	2
Chaudhuri et al. [25]	*Vascular*	2021	2013–2020	Prospectively Collected, Retrospective Analysis	1
Valdivia et al. [26]	*Vascular*	2022	2010–2019	Prospectively Collected, Retrospective Analysis	7
Bordet et al. [27]	*J Vasc Surg*	2023	2012–2020	Retrospective	1
Arko et al. [28]	*J Vasc Surg*	2023	2012–2015	Prospective	43
Abdel-Hadi et al. [29]	*J Vasc Interv Radiol*	2023	2017–2021	Retrospective	1
Valdivia et al. [30]	*Ann Vasc Surg*	2024	2010–2019	Prospectively Collected Retrospective Analysis	9
Sivak et al. [31]	*Bratisl Med J*	2024	2018–2021	Retrospective	1

**Table 2:** Cohort Demographics, Type of Repair (Index, Revision), Urgency of Repair (Intact, Rupture), and Endograft Type.

	Number of Patients	Sex (Male/Female)	Age	Index EVAR	Revision (EL Ia/Device Migration)	Intact/Rupture AAA	Endograft Type
Avci et al. [19]	11	8/3	77 (59–88)	0	11	11/0	Talent, Excluder, AneuRx
Perdikides et al. [20]	13	13/0	73 (62–82)	13	0	13/0	Endurant, Zenith
Jordan Jr WD et al. [15]	319	238/81	74.1 ± 8.2	242	77	315/4	Excluder, Zenith, Endurant, AneuRx, Talent, Other
Jordan Jr WD et al. [8]	100	80/20	73 ± 8	73	27	NA	Excluder, Zenith, Endurant, AneuRx, Talent, Other
Goudeketting et al. [21]	51	38/13	75 (53–78)	31	20	50/1	Endurant, Talent, Valiant, Zenith, Excluder
Giudice et al. [22]	17	NA	NA	9	8	17/0	Endurant
Valdivia et al. [23]	46	43/3	74.4 ± 8.1	46 [Therapeutic for Intraoperative EL Ia (22), Prophylactic (24)]	0	45/1	Endurant, Incraft
Ho et al. [24]	31	24/7	78.4–80.2	20 [Therapeutic for Intraoperativie EL Ia (10), Prophylactic (10)]	11	31/0	Endurant, Excluder
Chaudhuri et al. [10]	41	33/8	76.8 ± 8.9	35	6	36/5	Zenith Flex, Zenith Alpha, Endurant, Excluder C3
Chaudhuri et al. [25]	84	76/8	73.7 ± 7.8	84	0	84/0	Zenith Flex, Zenith Alpha, Zenith Aorto-Uni-Iliac, Endurant, Exluder, Incraft
Valdivia et al. [26]	221	184/37	75.6 (8.3)	175	46	213/8	Endurant, Zenith, Excluder, Incraft, E-tegra
Bordet et al. [27]	18	18/0	74 ± 7	18	0	18/0	Endurant
Arko et al. [28]	70	51/19	71.3 ± 8.1	70	0	70/0	Endurant
Abdel-Hadi et al. [29]	42	NA	NA	29	13	NA	NA
Valdivia et al. [30]	76	62/14	79.5 ± 7.5	76	0	NA	Endurant, Zenith, Excluder, Incraft, E-tegra
Sivak et al. [31]	24	22/2	73 ± 6.8	24	0	24/0	Endurant

Footnote: NA: not available.

**Table 3:** Heli-FX Endoanchor Deployment Indications and Cohort Neck Proximal Neck Criteria.

Author	Heli-FX Endoanchor Deployment Criteria	Cohort Unfavorable Neck Criteria (Number of Patients)
Avci et al. [19]	EL Ia, Device Migration, EL Ia + Device Migration	El Ia (4), Device Migration (1), EL Ia + Device Migration (6)
Perdikides et al. [20]	Neck Length < 15 mm, Neck Diameter > 29 mm, Angulation >45°, Conical Configuration, Irregural Shape (tapered, hourglass, barrel, bulged)	Angulation (8), Conical (7), Wide (7), Short (5), Bulged (2), Barrel (1), Irregular (1)
Jordan Jr WD et al. [15]	Index EVAR [Hostile Neck (Neck Length <10 mm, Neck Diameter > 28 mm, Angulation >60°, Conical Configuration, Mural Thrombus or Calcium > 2 mm, Intraoperative EL Ia or Misdeployment)], Revision [EL Ia, Device Migration]	Index EVAR [Hostile Neck (186), Residual Intraoperative EL Ia (52), Misdeployed Endograft (4)], Revision [EL Ia (45), Device Migration + EL Ia (21), Device Migration (11)]
Jordan Jr WD et al. [8]	Index EVAR [Hostile Neck, Intraoperative EL Ia or Misdeployment), Revision [EL Ia, Device Migration]	Index EVAR [Hostile Neck (63)], Revision [Hostile Neck (20)]
Goudeketting et al. [21]	Neck Length < 10 mm, Neck Diameter > 28 mm, Angulation >60°, Mural Thrombus or Calcium >2 mm thickness or <50% circumference, Conical Configuration)	Hostile Neck (48), Wide Neck (12),
Giudice et al. [22]	Hostile Neck Characteristics (Angulation >60o, Conical Configuration, Significant Thrombus or Calcium)	Angulation (3), Significant Thrombus (8), Conical Configuration (4)
Valdivia et al. [23]	Hostile Neck Characteristics (Angulation >60°, Conical Configuration, Significant Thrombus or Calcium, Neck Length < 10 mm, Neck Diameter > 32 mm)	Index EVAR [Hostile Neck (186), Residual Intraoperative EL Ia (52), Misdeployed Endograft (4)], Revision [EL Ia (45), Device Migration + EL Ia (21), Device Migration (11)]
Ho et al. [24]	Hostile Neck Characteristics, Residual Intraoperative EL Ia, EL Ia	Hostile Neck (10), Intraoperative EL Ia (11), Therapeutic EL Ia (10)
Chaudhuri et al. [10]	Angulation > 60°, EL Ia	Angulation >60° (35), Therapeutic EL Ia (6)
Chaudhuri et al. [25]	NR	Conical Configuration (22)
Valdivia et al. [26]	Hostile Neck [Neck Length < 10 mm + Angulation > 60° or >2 mm or >50% circumference thrombus or >50% circumference calcification or Conical Configuration or Neck Diameter > 28 mm or Asymmetric Neck Bulges]	Conical Configuration (82), Bulge Shape (12)
Bordet et al. [27]	Neck Length < 15 mm	Neck Length < 15 mm (18)
Arko et al. [28]	Index EVAR [Hostile Neck, Intraoperative EL Ia or Misdeployment), Revision [EL Ia, Device Migration]	NR
Abdel-Hadi et al. [29]	Hostile Neck (Neck Length < 10 mm, Angulation > 60°, Neck Diameter > 28 mm)	Hostile Neck (28)
Valdivia et al. [30]	Hostile Neck [Neck Length < 10 mm + Angulation >60° or >2 mm or >50% circumference thrombus or >50% circumference calcification or Conical Configuration or Neck Diameter > 28 mm or Asymmetric Neck Bulges]	Neck Length ≥ 4 and <7 mm (17), Neck Length ≥ 7 and <10 mm (59)
Sivak et al. [31]	Neck Length < 15 mm, Neck Diameter > 28 mm, Angulation >60°, Reverse Taper Configuration, >50% Circumference Calcification/Thrombus	Neck Length < 15 mm (13), Angulation >60° (7), Wide Neck (2)

Footnote: NR; Not Reported.

**Table 4:** Number of Heli-FX Endoanchors per procedure and Proximal Aortic Neck Characteristics.

Author	Number of Heli-FX Endoanchors	Aneurysm Diameter (mm)	Neck Diameter (mm)	Neck Leght (mm)
Avci et al. [19]	6 (4–9)	NR	NR	NR
Perdikides et al. [20]	4 (3–10)	56 (50–98)	32 (26–34)	16 (8–35)
Jordan Jr WD et al. [15]	Index EVAR: 5 (4–6), Revision: 7 (5–8)	58 ± 13 (51–63)	27 ± 4 (25–30)	16 ± 13 (7–23)
Jordan Jr WD et al. [8]	5.3 ± 1.8	58 ± 16	27 ± 5	16 ± 13.7
Goudeketting et al. [21]	Index EVAR: 6 (5–7), Revision: 6 (5–9)	63.7 (57.3–71)	27.7 (23, 30.1)	9 (5,18)
Giudice et al. [22]	5 (4–10)	60 (43–88)	29.5	10.7
Valdivia et al. [23]	6 ± 1.9	58.2 ± 8	25.5	8 (3–38)
Ho et al. [24]	8.3–10	68.7	NR	NR
Chaudhuri et al. [10]	6 ± 2	71.6 ± 16	24.5 ± 4.3	19.18 ± 12
Chaudhuri et al. [25]	7 ± 2	65.1 (12.2)	25 (22–31)	19 (13–28)
Valdivia et al. [26]	6 ± 3	63 ± 15.1	26 (7)	13 (10)
Bordet et al. [27]	4–8	54 (52–61)	25 (22–26)	8 (6–12)
Arko et al. [28]	NR	NR	NR	NR
Abdel-Hadi et al. [29]	7 (3–10)	NR	NR	NR
Valdivia et al. [30]	6 (3)	57 (12.8)	24 (6)	8 (3)
Sivak et al. [31]	7 (4–12)	NR	22.9 (20–28.2)	15 (7–46)

Footnote: NR—Not Reported.

**Table 5:** Follow-Up duration, Technical and Clinical Succes, Residual EL Ia.

Author	Technical Success	Procedural Success	Residual Intraoperative EL Ia	Freedom from EL Ia (Maximum FU)	Follow-Up (months)
Avci et al. [19]	100% (Revision only)	82% (Revision only)	2	100%	10 (3–18)
Perdikides et al. [20]	85% (Index EVAR only)	85% (Index EVAR only)	2	100%	7 (2–17)
Jordan Jr WD et al. [15]	95% (Index EVAR: 96.3%, Revision: 90.9%)	87.5% (Index EVAR: 89.7%, Revision: 80.5%)	29 (Index EVAR: 19, Revision: 10)	100%	9.3 ± 4.7
Jordan Jr WD et al. [8]	93% (Index EVAR: 95%, Revision: 89%)	89% (Index EVAR: 92%, Revision: 81%)	9 (Index EVAR: 3, Revision: 3)	Index EVAR 95% (95% CI 89–100%), Revision 77% (95% CI 60–95%)	18 ± 4
Goudeketting et al. [21]	99% (Index EVAR: NR, Revision: NR)	80% (Index EVAR: 87%, Revision: 70%)	10 (Index EVAR: 4, Revision: 6)	Index EVAR 88.9% (95% CI 76.9–100%), Revision 84% (95% CI 62.4–100%)	23.9 (13.4, 35.6)
Giudice et al. [22]	100% (Index EVAR: 100%, Revision: 100%)	100% (Index EVAR:100%, Revision: 100%)	0	100%	13 (4–39)
Valdivia et al. [23]	97.8% (Index EVAR only)	NR	9 (Index EVAR only)	100%	15 ± 12.9
Ho et al. [24]	100% (Index EVAR: 100%, Revision: 100%)	Revision (45.4%), Therapeutic for Intraoperative EL Ia during index EVAR (100%), Prophylactic (100%)	5 (Index EVAR: 0, Revision:5)	NR	9.4–13.6
Chaudhuri et al. [10]	100% (Index EVAR:100%, Revision:100%)	97.6% (Index EVAR:100%, Revision: 84%)	0	97.5%	18.5 (13.3–23.9)
Chaudhuri et al. [25]	NR	NR	1 (Index EVAR only)	100%	28.5 (12–43)
Valdivia et al. [26]	94.3% (Index EVAR: NR, Revision: NR)	89.1% (Index EVAR: NR, Revision: NR)	23 (Index EVAR:NR, Revision: NR)	Index EVAR 96%, Revision 86%	27 (12–48)
Bordet et al. [27]	100% (Index EVAR only)	94% (Index EVAR only)	1 (Index EVAR only)	100%	23 (19–33)
Arko et al. [28]	NR	NR	NR	NR	60
Abdel-Hadi et al. [29]	98% (Index EVAR:100%, Revision: 92.4%)	96% (Index EVAR: 100%, Revision: 94.7%)	2 (Index EVAR: 0, Revision: 2)	93%	38 (2–71)
Valdivia et al. [30]	86.8% (Index EVAR only)	86.8% (Index EVAR only)	10 (Index EVAR only)	84%	40.5 (12–61)
Sivak et al. [31]	100% (Index EVAR only)	100% (Index EVAR Only)	0	95.8%	22.5 (2–31.5)

Footnote: EL Ia; Endoleak Ia.

**Table 6:** Postoperative Proximal Aortic Neck-Related Outcomes.

Author	Secondary Interventions for Proximal Neck Complications (EL Ia, Migration, AND)	New Postoperative EL Ia (Maximum FU)	Postoperative Neck Diameter (mm)
Avci et al. [19]	0	0	>5 Decrease
Perdikides et al. [20]	0	0	32 (26–34)
Jordan Jr WD et al. [15]	8 (Index EVAR: 1, Revision: 7) (Reintervention for EL Ia: 7, Reintervention for Device Migration: 1)	0	NR
Jordan Jr WD et al. [8]	4 (Index EVAR: 1, Revision 3) (Reintervention for EL Ia: 4, Reintervention for Device MigrationJ	1	NR
Goudeketting et al. [21]	5 (Index EVAR: 1, Revision: 4) (Reintervention for EL Ia: 5, Reintervention for Device Migration: 0)	6	2.5 (0.6, 3.8) Increase
Giudice et al. [22]	0	0	NR
Valdivia et al. [23]	0	0	26.5
Ho et al. [24]	1 (Index EVAR: 0, Revision: 1) (Reintervention for EL Ia: 1, Reintervention for Device Migration: 0)	0	NR
Chaudhuri et al. [10]	0	0	3.2 ± 3.7 Increase
Chaudhuri et al. [25]	0	0	4.6 (5.7) Increase
Valdivia et al. [26]	10 (Index EVAR: 6, Revision: 4) (Reintervention for EL Ia: NR, Reintervention for Device Migration: NR)	3	NR
Bordet et al. [27]	0	0	No Change
Arko et al. [28]	3 (Index EVAR only) (Reintervention for EL Ia: 3)	9	NR
Abdel-Hadi et al. [29]	5 (Index EVAR: NR, Revision: NR) (Reintervention for EL Ia: 5, Reintervention for Device Migration: 0)	NR	NR
Valdivia et al. [30]	5 (Index EVAR only) (Reintervention for EL Ia: 5, Reintervention for Device Migration: 0)	1	NR
Sivak et al. [31]	1 (Index EVAR Only) (Reintervention for EL Ia: 1, Reintervention for Device Migration: 0)	1	NR

Footnote: AND—Aortic neck dilatation, NR—Not reported.

## Data Availability

Data are available after reasonable request from the corresponding author.

## Supplementary Materials

The following supporting information can be downloaded at: https://www.mdpi.com/article/10.3390/medicina62010040/s1, Table S1: PICO model. Table S2: Search strategy per protocol. Table S3: Preferred Reporting Items for Systematic reviews and Meta-Analyses extension for Scoping Reviews (PRISMA-ScR) Checklist. Figure S1:ROBINS-I tool for risk of bias assessment.
