# Supplementary material for: The Prophylactic and Therapeutic Use of the Heli-FX EndoAnchor System in Patients Undergoing Endovascular Aortic Aneurysm Repair—A Scoping Review
Source: Medicina (Kaunas). 2025 Dec 25;62(1):40. doi: 10.3390/medicina62010040 (PMC12842687; doi:10.3390/medicina62010040)
Supplement: Supplementary file 1 [file medicina-62-00040-s001.zip › Supplementary Table S1. PICO.docx]

| P | Patient, population or problem | Patients with an infrarenal abdominal aortic aneurysm undergoing EVAR |
| --- | --- | --- |
| I | Intervention, prognostic factor or exposure | EVAR with concomitant Heli-FX Endoanchor deployment |
| C | Comparison of intervention | - |
| O | Outcome you would like to measure or achieve | EL Ia, Device Migration, AND |
|  | What type of question are you asking? | -Is the deployment of the Heli-FX Endoanchor system during EVAR associated with a lower risk for device migration?  - Is the deployment of the Heli-FX Endoanchor system during EVAR associated with a lower risk of AND?  - Is the deployment of the Heli-FX Endoanchor system during EVAR associated with a lower risk of EL Ia? |
|  | Type of study you want to find | Observational studies (Randomized, Non-randomized) |

**Supplementary Table S1. PICO model**

Footnote: EVAR; Endovascular aortic aneurysm repair, EL; Endoleak, AND; Aortic-Neck Dilatation
