# Supplementary material for: The Prophylactic and Therapeutic Use of the Heli-FX EndoAnchor System in Patients Undergoing Endovascular Aortic Aneurysm Repair—A Scoping Review
Source: Medicina (Kaunas). 2025 Dec 25;62(1):40. doi: 10.3390/medicina62010040 (PMC12842687; doi:10.3390/medicina62010040)
Supplement: Supplementary file 1 [file medicina-62-00040-s001.zip › Supplementary Table S2. Search Strategy.docx]

**Supplementary Table S2. Search strategy per protocol**

| PUBMED  Hits: 34 | (((((((((abdominal aortic aneurysm) OR (abdominal aortic aneurysm[MeSH Terms])) OR ((AAA[MeSH Terms]) OR (AAA))) OR ((infrarenal abdominal aortic aneurysm) OR (infrarenal abdominal aortic aneurysm[MeSH Terms]))) OR ((endovascular aortic aneurysm repair) OR (endovascular aortic aneurysm repair[MeSH Terms]))) OR ((endovascular aneurysm repair) OR (endovascular aneurysm repair[MeSH Terms]))) OR ((EVAR) OR (EVAR[MeSH Terms]))) AND ((((Heli-FX Endoanchor) OR (Heli-FX Endoanchor[MeSH Terms])) OR ((Aptus Endoanchors) OR (Aptus Endoanchors[MeSH Terms]))) OR ((HeliFX Endoanchors[MeSH Terms]) OR (HeliFX Endoanchors)))) AND (((((((endoleak Ia) OR (endoleak Ia[MeSH Terms])) OR ((EL Ia) OR (EL Ia[MeSH Terms]))) OR ((device migration) OR (device migration[MeSH Terms]))) OR ((endograft migration) OR (endograft migration[MeSH Terms]))) OR ((aortic neck dilatation) OR (aortic neck dilatation[MeSH Terms]))) OR ((proximal neck dilatation) OR (proximal neck dilatation[MeSH Terms]))) |
| --- | --- |
| SCOPUS  Hits: 52 | "abdominal aortic aneurysm" OR "AAA" OR "infrarenal abdominal aortic aneurym" OR "endovascular aortic aneurysm repair" OR "endovascular aneurysm repair" OR "EVAR" AND "Heli-FX Endoanchor" OR "Aptus Endoanchors" OR "HeliFX endoanchors" AND "endoleak Ia" OR "EL Ia" OR "device migration" OR "endograft migration" OR "aortic neck dilatation" OR "proximal neck dilatation" |
| CENTRAL  Hits: 2 | #1 abdominal aortic aneurysm  #2 [mh "abdominal aortic aneurysm"]  #3 AAA  #4 [mh "AAA"]  #5 infrarenal abdominal aortic aneurysm  #6 [mh "infrarenal abdominal aortic aneurysm"]  #7 endovascular aortic aneurysm repair  #8 [mh "endovascular aortic aneurysm repair"]  #9 EVAR  #10 [mh "EVAR"]  #11 Heli-FX Endoanchor  #12 [mh "Heli-FX Endoanchor"]  #13 Aptus endoanchors  #14 [mh "Aptus endoanchors"]  #15 HeliFX endoanchors  #16 [mh "HeliFX endoanchors"]  #17 endoleak Ia  #18 [mh "endoleak Ia"]  #19 EL Ia  #20 [mh "EL Ia"]  #21 device migration  #22 [mh "device migration"]  #23 endograft migration  #24 [mh "endograft migration"]  #25 aortic neck dilatation  #26 [mh "aortic neck dilatation"]  #27 proximal neck dilatation  #28 [mh "proximal neck dilatation"]  #29 {OR #1-#10}  #30 {OR #11-#16}  #31 {OR #17-#28}  #32 {AND #29-#31} |

Total Hits: 88

Duplicate Screening:

Automatic Tool Duplicate Removal: 78 (10 duplicates excluded)

By-Hand Duplicate Removal: 68 (10 duplicates excluded)

Title-Abstract Screening: 39 (29 excluded)

Full-text Screening: 16 (23 excluded)
