# Supplementary figures and images for: The Prophylactic and Therapeutic Use of the Heli-FX EndoAnchor System in Patients Undergoing Endovascular Aortic Aneurysm Repair—A Scoping Review
Source: Medicina (Kaunas). 2025 Dec 25;62(1):40. doi: 10.3390/medicina62010040 (PMC12842687; doi:10.3390/medicina62010040)

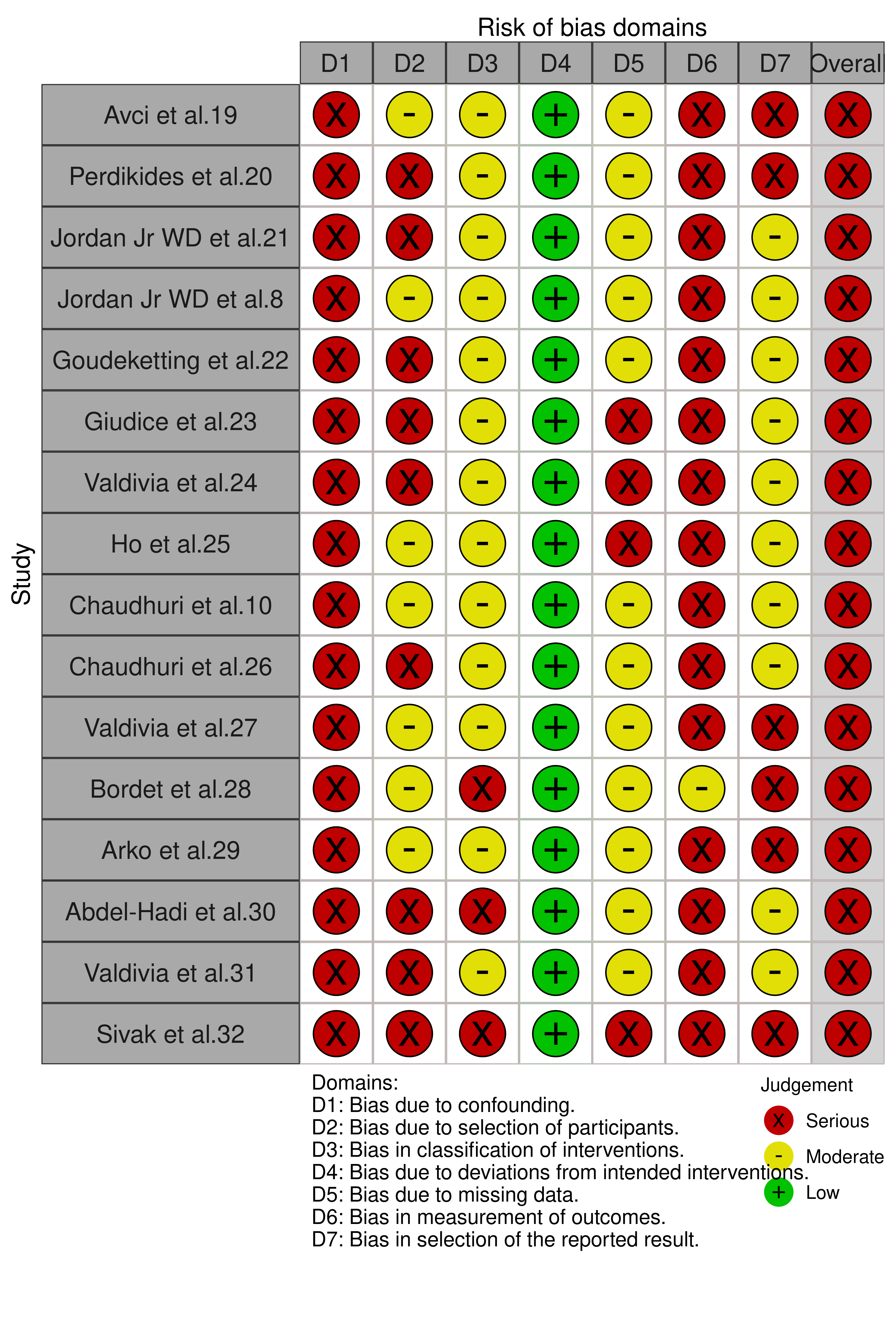

Supplement: Supplementary file 1 [file medicina-62-00040-s001.zip › Figure S1.png]
